# Prediction of Occult Cervical Lymph Node Metastasis in Bone-Invasive pT4a cN0 Oral Squamous Cell Carcinoma in Relation to Tumor Size: A Retrospective Observational Cohort Study

**DOI:** 10.3390/cancers17183044

**Published:** 2025-09-18

**Authors:** Friedrich Mrosk, Victoria Vertic, Maximilian Richter, Erin Sprünken, Lukas Mödl, Jan Oliver Voss, Anna Sofroniou, Carsten Rendenbach, Max Heiland, Steffen Koerdt

**Affiliations:** 1 Department of Oral and Maxillofacial Surgery, Charité—Universitätsmedizin Berlin, Freie Universität Berlin and Humboldt-Universität zu Berlin, Augustenburger Platz 1, 13353 Berlin, Germany; victoria.pereira-vertic@charite.de (V.V.); maximilian.richter@charite.de (M.R.); jan.voss@charite.de (J.O.V.); anna.sofroniou@charite.de (A.S.); carsten.rendenbach@charite.de (C.R.); max.heiland@charite.de (M.H.); steffen.koerdt@charite.de (S.K.); 2 Institute of Biometry and Clinical Epidemiology and Berlin Institute of Health, Charité—Universitätsmedizin Berlin, Freie Universität Berlin and Humboldt-Universität zu Berlin, Chariteplatz 1, 10117 Berlin, Germany; erin.spruenken@charite.de (E.S.); lukas.moedl@charite.de (L.M.)

**Keywords:** oral squamous cell carcinoma, bone invasion, T4a, cervical lymph node metastasis

## Abstract

**Objective:** The T classification of oral squamous cell carcinoma (OSCC) is linear according to the tumor size, excluding T4a by its criteria of invasion into adjacent structures, such as cortical bone. This may lead to the upstaging of otherwise small tumors. The objective was to analyze patients with OSCC and negative nodal staging to assess the impact of T-staging with tumor size on the incidence of occult cervical lymph node metastasis (CLNM) and regional neck failure. **Methods:** This retrospective cohort study included patients with OSCC and clinically negative necks (cN0), treated surgically between 2010 and 2024. All T4a OSCC classified due to bone invasion were additionally reclassified into T1–T3 based on size and depth of invasion according to the current staging manual. The primary endpoint of this study was the association between OSCC stratified by T-stage and tumor size as well as the presence of occult CLNM. **Results:** A total of 642 patients were included, with an overall occult CLNM rate of 20.2%. Bone invasion in T1-sized tumors was significantly associated with occult CLNM (OR 6.38, 95% CI: 1.48–27.42), whereas no such association was observed in T2 or T3 tumors (OR 0.80, 95% CI: 0.37–1.73; and OR 0.77, 95% CI: 0.37–1.62, respectively). Additionally, in T1–T2 tumors, bone invasion did not correlate with worse survival outcomes. **Conclusions:** Bone invasion was not significantly associated with occult CLNM in T2-3 sized OSCC, suggesting that the prognostic relevance is size-dependent. These findings question the uniform upstaging to T4a and support a more differentiated approach, potentially enabling neck management de-escalation in selected early-stage cases.

## Simple Summary

This retrospective cohort study analyzed 642 patients with oral squamous cell carcinoma (OSCC) and clinically negative necks (cN0) treated surgically between 2010 and 2024. Tumors initially staged as T4a due to bone invasion were reclassified to T1–T3 based on size and depth of invasion. The overall rate of occult cervical lymph node metastasis (CLNM) was 20.2%. Bone invasion significantly increased occult CLNM risk in T1-sized tumors (OR 6.38, 95% CI: 1.48–27.42), but not in T2–T3 tumors. Moreover, bone invasion in T1–T2 tumors was not associated with worse survival. These findings suggest that the prognostic relevance of bone invasion is size-dependent. Routine upstaging to T4a may therefore overestimate risk in certain cases, and a more differentiated staging approach could support de-escalation of neck management in selected early-stage OSCC patients.

## 1. Introduction

The oncological prognosis of oral squamous cell carcinoma (OSCC) varies according to individual histopathological risk profiles, which has important clinical and therapeutic implications. The primary classification system used is the TNM staging, with the T stage describing the local extent of the tumor based on size and depth of invasion (DOI) [1]. In general, tumor progression is linear from T1 to T3 based on these parameters. However, T4a tumors deviate from this pattern, as they are classified solely based on invasion into adjacent anatomical structures such as the bone, regardless of size or DOI. This means that even a small tumor typically categorized as T1 may be upstaged to T4a if cortical bone invasion is present [2]. This reclassification comes along with clinical implications.

In patients with clinically negative necks (cN0), the risk of occult cervical metastasis usually remains high with up to 40%, prompting the recommendation for some form of neck management [3]. In addition, this risk of occult metastasis correlates, in part, with higher T stages [4,5,6]. Nevertheless, recent randomized controlled trials and current guidelines support surgical de-escalation of the neck in early-stage diseases without initial evidence of cervical lymph node metastasis (CLNM), typically via sentinel lymph node biopsy [7,8,9,10]. Here, stages T1–2 are usually considered “early-stage,” while T3–4 are grouped as “advanced-stage” diseases [10]. This de-escalating approach maintains oncological safety while minimizing surgical morbidity [11]. With the growing importance of immunotherapy in multimodal OSCC treatment, preserving immunocompetence through surgical de-escalation has become of major interest [12]. This raises a critical question about the tumor classification: does bone invasion alone justify upstaging and the accompanying need for more extensive neck management? Accordingly, the aim of this study is to analyze patients with OSCC and negative nodal staging to assess the impact of T-staging with tumor size on the incidence of occult metastasis and regional neck failure.

## 2. Methods

### 2.1. Study Design

This retrospective, monocentric, observational cohort study was conducted at the Department of Oral and Maxillofacial Surgery, Charité—Universitätsmedizin Berlin and was approved by the local ethics committee (reference number: EA2-077-20). Patients with histologically confirmed squamous cell carcinoma of the oral cavity and a clinically negative neck (cN0) were identified from the institutional cancer registry. All included patients underwent primary surgical resection with negative margins (R0) between January 2010 and December 2024. All patients with evidence of bone invasion received segmental bone resection. Only pT4a OSCC cases classified based on the presence of bone invasion were included. Tumors staged as pT4a due to invasion of adjacent structures other than bone—such as deep muscles, the maxillary sinus, or skin—were excluded. Additional exclusion criteria included synchronous secondary carcinomas, different head and neck cancer locations such as oropharyngeal SCC or any prior history of head and neck cancer.

Parameters assessed in this study included age, sex, tumor-specific characteristics including tumor location and histopathological risk factors, treatment modality, and follow-up events such as locoregional failure, distant metastasis, and mortality. All tumors were retrospectively restaged according to the current 8th edition of the AJCC Cancer Staging Manual [1]. To allow comparisons based on the tumor size and depth of invasion, all pT4a OSCC tumors were additionally reclassified into the corresponding T1, T2, or T3 categories according to size and DOI.

The primary endpoint of this study was the association between OSCC stratified by T-stage and tumor size as well as the presence of occult CLNM. Secondary endpoints included regional failure quantified by neck control rate (NCR), overall survival (OS) and recurrence-free survival (RFS).

### 2.2. Statistical Analysis

Descriptive statistics were presented as absolute and relative frequencies for categorical variables, and as mean ± standard deviation (SD) for continuous variables. Univariate and multivariate logistic regression models were employed to assess the association between epidemiological and histopathological risk factors and the presence of occult cervical lymph node metastasis (CLNM), with results reported as odds ratios (OR) and corresponding 95% confidence intervals (95% CI). Model selection for the logistic regression analyses was performed using the Akaike Information Criterion (AIC) to identify the best-fitting models. Kaplan–Meier survival analysis was used to estimate OS, RFS and NCR. OS was defined as the time from diagnosis to death from any cause; RFS as the time to locoregional recurrence, distant metastasis, or death; and NCR as the time to regional lymph node failure. Survival differences between groups were analyzed using the log-rank test. A *p*-value of <0.05 was considered statistically significant. All statistical analyses were conducted using R (version 4.5.1; R Foundation for Statistical Computing, Vienna, Austria). 

## 3. Results

### 3.1. Patient Cohort Characteristics

Overall, 642 patients could be included, of whom 117 (18.2%) were initially staged as pT4a. The baseline characteristics of both the overall cohort and the pT4a cohort with its size reclassification are shown in Table 1. Tumor locations were as follows: tongue in 183 (28.5%), floor of mouth in 164 (25.5%), lower jaw in 160 (24.9%), buccal mucosa in 53 (8.3%), upper jaw in 49 (7.6%), multiple regions in 27 (4.2%) and hard palate in 6 (0.9%) cases. Bone-invasive tumors presented at the lower jaw in 72 (61.5%), at the upper jaw in 21 (17.9%), the floor of mouth in 19 (16.2%) and at the hard palate in 5 (4.3%) cases. Patients with pT4a tumors, when classified based on tumor size if no bone invasion would be present, exhibited higher disease stages compared to the overall cohort. There was no association between tumor location in bone-invasive OSCC and the presence of occult CLNM.

Among the 642 patients included in the study, the majority (*n* = 593) underwent elective neck dissection (END), while a smaller subgroup (*n* = 49) was managed using sentinel lymph node biopsy (SLNB). SLNB was only performed in pT1-2 OSCC. The number of lymph nodes retrieved ranged from 1 to 81, with a mean yield of 25.5 nodes (95% CI: 24.2–26.8). Adjuvant therapy (AT) was administered in 151 cases (23.5%), whereas 491 patients (76.5%) did not receive additional treatment following surgery. From all bone-invasive tumors, only 70 (59.8%) received AT.

### 3.2. Cervical Lymph Node Metastasis

The overall rate of occult metastasis in the cohort was 20.2%. When stratified by tumor stage, patients with pT4 tumors showed a notably higher rate of occult metastasis at 29.9%. Table 2 is presenting the distribution of occult metastasis among T-stages and classification of pT4a patients according to the tumor size.

After adjustment for age, sex and histopathological risk factors, bone invasion in T1-sized tumors was significantly associated with occult CLNM (OR 6.38, 95% CI: 1.48–27.42). In contrast, for T2- and T3-sized tumors, the presence of bone invasion was not significantly associated with occult CLNM (T2: OR 0.80, 95% CI: 0.37–1.73 and T3: OR 0.77, 95% CI: 0.37–1.62).

**Figure 1:**
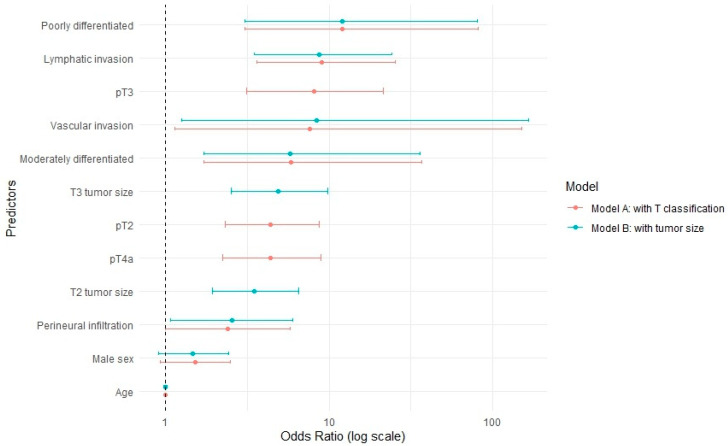
Graphical illustration of the multivariate logistic regression models including T-stage classification (A) and tumor size (B).

Table 3 and Figure 1 present two larger multivariate logistic regression models: Model A includes T-stage classification based on bone invasion, while the second model incorporates tumor size, including reclassified pT4a cases based on size. Model A (based on pT classification) demonstrated a better model fit with a lower AIC value (145.2) compared to Model B (based on tumor size), which had an AIC of 157.9.

### 3.3. Survival and Regional Failure

The 3-year OS for the entire cohort was 76.7% (95% CI: 72.7–80.9), while the RFS was 63.8% (95% CI: 59.3–68.6). During follow-up, only 36 (5.6%) patients presented with regional lymph node failure. The 3-year NCR was 92.2% (95% CI: 89.5–94.9).

**Figure 2:**
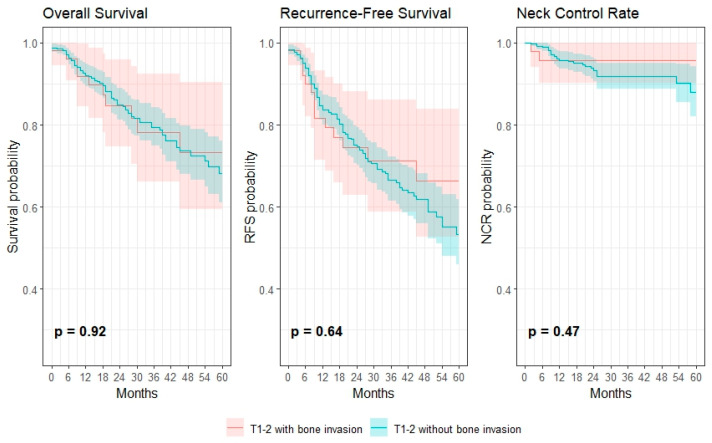
Kaplan–Meier survival analysis of the OS, RFS and NCR in relation to T1-2 tumors with and without bone invasion.

Patients with pT1 tumors had the most favorable prognosis, with a 3-year OS of 85.6% (95% CI: 80.5–91.0), NCR of 93.8% (95% CI: 90.2–97.5), and RFS of 72.1% (95% CI: 65.7–79.2). In contrast, pT3 tumors were associated with significantly poorer outcomes, with a 3-year OS of 55.4% (95% CI: 40.0–76.7), NCR of 80.6% (95% CI: 62.3–100.0), and RFS of 26.7% (95% CI: 12.3–58.0). Both pT2 (3-year OS of 70.8% (95% CI: 63.0–79.6), NCR of 89.0% (95% CI: 83.4–95.0), and RFS of 58.6% (95% CI: 50.4–68.1)) and pT4a (3-year OS of 74.7% (95% CI: 66.1–84.4), NCR of 96.0% (95% CI: 92.1–99.9), and RFS of 65.9% (95% CI: 57.0–76.2)) stages showed intermediate survival. Among patients with small tumors (T1–2 sized), bone invasion was not significantly associated with lower survival outcomes (see Figure 2). The 3-year OS, RFS and NCR with bone invasion were 78.1% (95% CI: 66.1–92.4), 71.2% (95% CI: 58.8–86.1) and 95.8% (95% CI: 90.2–100), respectively. For tumors without bone invasion, the 3-year OS, RFS and NCR with bone invasion were 79.5% (95% CI: 74.9–84.3), 66.6% (95% CI: 61.4–72.3) and 91.9% (95% CI: 88.8–95.2), respectively. AT was not significantly associated with differences in 3-year OS (74.5%, 95% CI: 62.2–86.8 vs. 69.0%, 95% CI: 52.7–85.3), 3-year RFS (69.7%, 95% CI: 58.3–81.1 vs. 58.4%, 95% CI: 41.2–75.6) and 3-year NCR (100%, 95% CI: 0–100 vs. 93.7%, 95% CI: 87.6–99.8).

## 4. Discussion

In this study, we demonstrated that bone invasion appears to be associated with an increased risk of occult metastasis in OSCC, which according to size would be classified as pT1. However, this association was not present for pT2 or pT3 sized tumors, where bone invasion was not statistically significant different in regard to the prediction of occult CLNM or regional failure. To interpret these results, it is important to consider that only 9 patients with pT4a tumors could be classified to pT1 according to the tumor size. Therefore, these results need to be interpreted with care since they may underly sample size bias. In our pT-stage prediction model, pT2 tumors were even slightly stronger predictive than pT4a tumors, which is also reflective in the distribution of occult metastasis illustrated in Table 2.

Beyond these findings, the literature reflects a heterogeneous landscape regarding the prognostic significance of bone invasion. For example, Bittar et al. suggested pT4a classification, PNI, and tumor thickness were predictive for occult CLNM, though their study methodology was limited and potentially biased due to incomplete statistical modeling and incomplete case stratification [13]. More recent data from Dey et al. supported a statistically significant association between bone invasion and CLNM, but again, without tumor size stratification, their findings risk overestimating the role of bone invasion in small tumors [14]. Contrasting these results, other studies failed to establish an association between bone invasion and CLNM and instead identified the pattern of invasion as a more relevant predictor [15]. Similar to our results but focusing on the tumor volume, Lin et al. emphasized this parameter as a prognostic factor in T4a OSCC, associating larger tumors with worse recurrence and survival, yet they did not distinguish bone invasion as an isolated criterion, nor did they assess the prediction of occult CLNM specifically [16]. Interestingly, in studies focusing on upper gingival or mandibular tumors, predictors such as bucco-lingual tumor width, grade of differentiation, and tumor location were shown to be more influential than bone invasion in forecasting nodal involvement [17]. Moreover, Shaw et al. highlighted that tumor size, nodal involvement, and soft tissue invasion patterns held greater prognostic value than bone involvement alone for local recurrence [18].

One study assessing 323 OSCC patients stratified by tumor size and bone invasion status [19]. The authors found that, for tumors measuring less than 4 cm (T1-2), bone invasion was not significantly associated with overall disease progression, which aligns with our finding that bone invasion does not independently predict occult CLNM in T2-sized tumors. However, their subgroup analysis revealed that T2 mandibular tumors with both buccal and lingual cortical bone invasion demonstrated significantly worse prognosis. Notably, this finding likely reflects the effect of extensive local infiltration rather than regional metastatic spread, as their outcome measure disease-free survival included local, regional, and distant recurrence collectively. This distinction is essential, as it suggests that the adverse prognostic effect observed may be more related to local control challenges rather than nodal disease, which our study specifically targeted. Therefore, their findings do not contradict our conclusion that bone invasion alone and particularly in smaller tumors may not necessitate more aggressive neck management.

Ebrahimi et al. further differentiated cortical versus medullary bone invasion [20]. While medullary involvement was indeed associated with worse disease-free survival, it was largely due to an increased risk of distant, but not regional metastasis. This highlights the complexity of interpreting the prognostic implications of bone invasion and the need for precise pathological subclassification.

In our cohort, the lack of independent prognostic significance of bone invasion aligns with findings that question the uniform upstaging to T4a solely on the basis of bone infiltration. These results advocate for a more nuanced interpretation of pT4a classification in OSCC. In particular, they call for caution when assigning pT4a status in smaller tumors, as this may lead to overtreatment, especially in patients who might otherwise qualify for less invasive neck management such as SLNB. This study is the first, to our knowledge, to systematically evaluate bone invasion in the context of tumor size reclassification and its direct impact on occult CLNM. Notably, no prospective SLNB trials to date have provided specific evidence on small T4a tumors with bone invasion, leaving a critical gap in clinical guidance for this subgroup.

Nevertheless, several limitations must be mentioned. First, the retrospective and monocentric nature of our study limits the generalizability of the results. In particular, the small number of reclassified T1-sized tumors with bone invasion warrants caution in overinterpreting these findings. Furthermore, bone invasion was treated as a binary variable without differentiation between cortical and medullary involvement, which may additionally underestimate its biological heterogeneity. On the other hand, the study benefits from a large, well-defined cohort and robust statistical modeling.

## 5. Conclusions

While our study demonstrates that bone invasion is significantly associated with an increased risk of occult CLNM in OSCC tumors of T1 size, this association does not persist in tumors of T2 or T3 size, where bone invasion does not offer additional predictive value for occult metastasis or regional failure. These findings challenge the current uniform upstaging of all bone-invasive tumors to pT4a and suggest that the prognostic relevance of bone invasion may be size-dependent. While the pathological T classification outperformed tumor size alone in predictive modeling, it should not be applied as a strictly linear risk gradient. In smaller tumors, particularly those otherwise qualifying as early-stage disease, assigning pT4a status based solely on bone invasion may lead to overtreatment and unnecessary intensification of neck management. Therefore, these findings support a more differentiated approach to T4a OSCC and raise the potential for surgical de-escalation, such as SLNB, in carefully selected patients. However, prospective studies, including trials incorporating SLNB in small bone-invasive OSCCs, are needed to validate these implications and guide future directions.

## Figures and Tables

**Table 1:** Baseline characteristics of the study cohort. The T-stage for pT4a patients refers to the assigned classification in absence of bone-invasion.

	All (*n* = 642, %)	pT4a (*n* = 117, %)
Age (mean ± SD)	64.5 ± 12.2	66.7 ± 11.7
Sex		
Female	266 (41.4)	45 (38.5)
Male	376 (58.6)	72 (61.5)
T-stage		
pT1	275 (42.8)	9 (7.7)
pT2	193 (30.1)	44 (37.6)
pT3	57 (8.9)	64 (54.7)
pT4a	117 (18.2)	-
N-stage		
pN0	512 (79.8)	82 (70.1)
pN+	130 (20.2)	35 (29.9)
Extracapsular spread		
Yes	23 (3.6)	9 (7.7)
No	619 (96.4)	108 (92.3)
Grade of differentiation		
I	87 (13.6)	4 (3.4)
II	490 (76.3)	98 (83.8)
III	65 (10.1)	15 (12.8)
Vascular infiltration		
Yes	10 (1.6)	2 (1.7)
No	632 (98.4)	115 (98.3)
Lymphatic infiltration		
Yes	26 (4.0)	7 (6.0)
No	616 (96.0)	110 (94.0)
Perineural invasion		
Yes	30 (4.7)	6 (5.1)
No	612 (95.3)	111 (94.9)

**Table 2:** Distribution of occult cervical lymph node metastasis regarding pT-stage and tumor size.

	Occult Cervical Lymph Node Metastasis
pT1	20/275 (7.3%)
pT2	52/193 (26.9%)
pT3	23/57 (40.4%)
pT4a	35/117 (29.9%)
T1 size	3/9 (33.3%)
T2 size	10/44 (22.7%)
T3 size	22/64 (34.4%)

**Table 3:** Multivariate logistic regression models for the prediction of occult lymph node metastasis including T-stage classification (A) and tumor size (B).

Predictor	OR (95% CI) (Model A)	OR (95% CI) (Model B)
Age (continous)	1 (0.98–1.02)	1 (0.98–1.01)
Sex		
Female	1 (Reference)	1 (Reference)
Male	1.53 (0.93–2.53)	1.56 (1.05–2.36)
T-stage		
pT1	1 (Reference)	
pT2	9.81 (1.59–53.39)	
pT3	11.89 (1.82–68.36)	
pT4a	7.22 (1.4–31.05)	
Tumor size		
T1 size		1 (Reference)
T2 size		4.02 (2.43–6.85)
T3 size		6.72 (3.86–11.97)
Vascular invasion	7.92 (1.18–156.9)	37.12 (6.88–687.75)
Lymphatic invasion	8.98 (3.55–25.14)	12.06 (5.16–31.51)
Perineural infiltration	2.41 (1–5.82)	4.04 (1.9–8.61)
Grade of differentiation		
Well differentiated	1 (Reference)	1 (Reference)
Moderately differentiated	5.81 (1.69–36.54)	11.17 (3.45–68.57)
Poorly differentiated	12.16 (3.06–82.02)	28.33 (7.93–181.44)
